# LPS-Induced Inflammation Potentiates the IL-1**β**-Mediated Reduction of LH Secretion from the Anterior Pituitary Explants

**DOI:** 10.1155/2013/926937

**Published:** 2013-07-17

**Authors:** Andrzej Przemysław Herman, Agata Krawczyńska, Joanna Bochenek, Elżbieta Dobek, Anna Herman, Dorota Tomaszewska-Zaremba

**Affiliations:** ^1^Polish Academy of Sciences, The Kielanowski Institute of Animal Physiology and Nutrition, 05-110 Jabłonna, Poland; ^2^The Academy of Cosmetics and Health Care, 13 Podwale Street, 00-252 Warsaw, Poland

## Abstract

Acting at the level of the brain, interleukin- (IL-)1**β** is considered to be one of the most potent downregulators of reproduction processes during immune/inflammatory challenge. IL-1**β** suppresses gonadotropin-releasing hormone (GnRH) secretion from the hypothalamus resulting in the inhibition of the luteinizing hormone (LH) release from the anterior pituitary (AP). However, the presence of IL-1**β** receptors in the AP suggests the possible direct action of this cytokine on LH secretion. The study was designed to determine the effect of IL-1**β** on the LH secretion from the AP explants collected from saline and LPS-treated ewes in the follicular phase. It was found that IL-1**β** suppressed (*P* ≤ 0.01) GnRH-stimulated LH release and LH**β** gene expression in AP explants in both groups. However, IL-1**β** action was more potent in the explants collected from LPS-treated animals. Pituitaries from LPS-treated animals were characterized by increased (*P* ≤ 0.01) IL-1 type I receptor and decreased (*P* ≤ 0.01) GnRH receptor gene expression level compared to the saline-treated group. IL-1**β** also affected the GnRH-R gene expression in explants collected from LPS-treated animals. Our results show that direct action of IL-1**β** on the pituitary gonadotropes could be one of the reasons of the reproductive processes disorders accompanying an inflammatory state.

## 1. Introduction

An immune/inflammatory challenge is considered as an important factor inhibiting the reproduction process in animals and human. The inflammation caused by peripheral administration of bacterial endotoxin-lipopolysaccharide (LPS) significantly decreases gonadotropin-releasing hormone (GnRH) and luteinizing hormone (LH) secretion [1–3]. These interconnections existing between the immune and the neuroendocrine systems are based on the mutual sharing of receptors and mediators [4]. Numerous *in vitro* and *in vivo* studies showed that the immune stress affects the GnRH/LH secretion by the central action of proinflammatory cytokines affecting the secretory activity of GnRH neurons in the hypothalamus [4–6]. One of the most potent and pleiotropic cytokines of the immune system is interleukin- (IL-)1*β* [7]. Its action at the level of the hypothalamus is considered as an important mechanism via inflammation downregulates GnRH/LH secretion [8–10]. However, the presence of IL-1 type I receptor (IL-1R1) in the pituitary [11, 12] suggests that antigonadotropic action of IL-1*β* could be more complex and may occur also at the level of this gland.

The present study was designed to determine the effect of IL-1*β* on the LH secretion from the anterior pituitary (AP) explants. Conducting *ex vivo* experiment on the pituitary explants instead of more popular primary cell culture lets us better imitate the reaction of AP under *ex vivo* conditions, because without dispersion the AP cells preserved many of their intercellular connections. Moreover, in the present studies, the pituitary explants were collected form saline and LPS-treated ewes. This should allow answering the questions whether the pituitaries kept their “memory” of the events triggered by LPS exposure and if that “immunological” status of animals can modulate the pituitary response on IL-1*β*.

## 2. Materials and Methods

### 2.1. Animals

The studies were performed on 3-year-old Blackhead ewes during the reproductive season (September-October). The animals were maintained indoors in individual pens and exposed to natural daylight. All ewes were healthy; their condition was continuously monitored by a qualified veterinarian. The ewes were well adapted to the experimental conditions and always had visual contact with neighbouring ewes, even during the experimental period, to prevent stress due to social isolation. The animals were fed a constant diet of commercial concentrates with hay and water available ad libitum.

The ewes were synchronized by Chronogest CR method (Merck Animal Health, Boxmeer, The Netherlands) using an intravaginal sponge impregnated with 20 mg of synthetic progesterone-like hormone. All ewes had Chronogest CR sponges placed for 14 days. Following sponge removal, the ewes received an intramuscular injection of 500 iu pregnant mare's serum gonadotropin (PMCS) (Merck Animal Health, Boxmeer, The Netherlands). The experimental procedure was started 24 h following PMSG injection.

All procedures on animals were performed with the consent of the Local Ethics Committee of the Warsaw Agricultural University.

## 3. Experimental Procedures

### 3.1. Inducing Immune Stress in the Experimental Animals

Ewes (*n* = 12) were randomly assigned to two experimental groups: control (*n* = 6) and LPS-treated groups (*n* = 6). In treated ewes, an innate immune system response was activated by injection of LPS (400 ng/kg of body weight) from *E. coli* 055:B5 (Sigma-Aldrich, St. Louis, MO, USA) dissolved in saline (0.9% w/v NaCl) (Baxter, Deerfield, IL, USA) at a concentration of 10 mg/L intravenously (i.v.) into jugular vein. The maximum volume of LPS solution (10 mg/L) injected to any animals never exceeds 2.5 mL. The control group received an equivalent to their body weight volume of NaCl.

### 3.2. Incubation of the AP Explants *Ex Vivo*

The animals from both groups were slaughtered by decapitation 2 hours after i.v. injection of LPS (*n* = 6) or saline (*n* = 6). The ovine brains were rapidly removed from the skulls and the APs were dissected. Immediately after slaughtering, the APs were divided into four fragments weighing from 50 to 60 mg which were transferred to 24-well plates (Becton Dickinson Labware, Franklin Lakes, NJ, USA). The *ex vivo *incubation of the explants was performed in medium 199 HEPES Modification (Sigma-Aldrich, St. Louis, MO, USA) suitable for cell culture with Penicillin-Streptomycin at the dose of 10 mL/L (Sigma-Aldrich, St. Louis, MO, USA) and incubated at 37°C, 87% O_2_, and 5% CO_2_. After the collection, all the tissues were preincubated for 1 h in 800 *μ*L of “pure” medium 199. During preincubation, the medium was changed for the fresh one four times every 15 min. The preincubation was performed to wash out blood and hormones remains from pituitary fragments. Then, the explants collected from each saline as well as LPS-treated ewe were divided into four experimental groups as follows: control—AP explants (*n* = 6) incubated in 600 *μ*L of medium 199; GnRH control—AP explants (*n* = 6) incubated in 600 *μ*L of medium 199 with GnRH (100 pmol/mL) (Sigma-Aldrich, St. Louis, MO, USA); IL-1*β*—AP explants (*n* = 6) incubated in 600 *μ*L of medium 199 with IL-1*β* (100 pg/mL) (Sigma-Aldrich, St. Louis, MO, USA); GnRH + IL-1*β*—AP explants (*n* = 6) incubated in 600 *μ*L of medium 199 with GnRH (100 pmol/mL); and IL-1*β* (100 pg/mL). The *ex vivo* experiment was carried out for 4 h. During 1 h of incubation, all explants were treated with 600 *μ*L of “pure” medium 199. The medium was changed to fresh three times every 20 min. After 1 h, all the AP explants were incubated in the experimental medium appropriate to each experimental group. The media were changed every 20 min for the fresh one and 600 *μ*L samples were collected. The dose of treatments, condition of incubation, and time of the experiment were previously optimized in the preliminary studies. After finished incubation, all tissues were frozen in liquid nitrogen and stored at −80°C until assay.

## 4. Assays

### 4.1. Radioimmunoassay for LH

The concentration of LH in medium was assayed by the RIA double antibody method using anti-ovine-LH and anti-rabbit-*γ*-globulin antisera and ovine standard (NIH-LH-SO18) as described by Stupnicki and Madej [13]. The sensitivity was 0.3 ng/mL, and intraassay and interassay coefficients of variation were 8.9% and 12.3%, respectively.

### 4.2. The Relative Gene Expression Assay

Total RNA from the AP tissues was isolated using NucleoSpin RNA II Kit (MACHEREY-NAGEL Gmbh and Co.; Düren, Germany) according to the manufacturer's instruction. The purity and concentration of isolated RNA were quantified spectrophotometrically by measuring the optical density at 230, 260, and 280 nm in a NanoDrop 1000 instrument (Thermo Fisher Scientific Inc., Waltham, USA). The RNA integrity was verified by electrophoresis using 1% agarose gel stained with ethidium bromide. Maxima First Strand cDNA Synthesis Kit for RT-qPCR (Thermo Fisher Scientific Inc., Waltham, USA) was used to prepare cDNA synthesis. As a starting material for this PCR synthesis, 2 *μ*g of total RNA was used.

Real-time RT-PCR was carried out using HOT FIREPol EvaGreen qPCR Mix Plus (Solis BioDyne, Tartu, Estonia) components and HPLC-grade oligonucleotide primers synthesised by Genomed (Poland). Specific primers for determining the expression of housekeeping genes and the genes of interest were designed using Primer 3 software. The sequences of the primers were as follows: LH*β* primers: 5′-AGATGCTCCAGGGACTGCT-3′ (forward) and 5′-TGCTTCATGCTGAGGCAGTA-3′ (reverse) (GenBank accession no. X52488), generated product size 184-bp; GnRH-R primers: 5′-TCTTTGCTGGACCACAGTTAT-3′ (forward) and 5′-GGCAGCTGAAGGTGAAAAAG-3′ (reverse) (GenBank accession no. NM-001009397), generated product size 150-bp; IL-1 receptor type I primers: 5′-GAGGAAGACTTTATCACAGTGGA-3′ (forward) and 5′-GGCTAAACAGGTAAATGGATGC-3′ (reverse) (GenBank accession no. NM_001206735.1), generated product size 120-bp; *β*-actin (ACTB) primers: 5′-CTTCCTTCCTGGGCATGG-3′ (forward) and 5′-GGGCAGTGATCTCTTTCTGC-3′ (reverse) (GenBank accession no. U39357), generated product size 168-bp; glyceraldehyde-3-phosphate dehydrogenase (GAPDH) primers: 5′-AGAAGGCTGGGGCTCACT-3′ (forward) and 5′-GGCATTGCTGACAATCTTGA-3′ (reverse) (GenBank accession no. NM-001034034), generated product size 134-bp; cyclophilin C (PPIC) primers: 5′-ACGGCCAAGGTCTTCTTTG-3′ (forward) and 5′-TATCCTTTCTCTCCCGTTGC-3′ (reverse) (GenBank accession no. NM-001076910), generated product size 131-bp. One tube contained 4 *μ*L PCR Master Mix (5x), 14 *μ*L RNase-free water, 1 *μ*L primers (0.5 *μ*L each, working concentration was 0.25 *μ*M), and 1 *μ*L cDNA template. The tubes were run on the Rotor-Gene 6000 (Qiagen, Duesseldorf, Germany). The following protocol was used: 95°C in 15 min for activating Hot Start DNA polymerase and finally the PCR including 30 cycles at 95°C in 10 sec for denaturation, 60°C in 20 sec for annealing, and 72°C in 10 sec for extension. After the cycles, a final melting curve analysis under continuous fluorescence measurements was performed to confirm the specificity of the amplification.

## 5. Data Analysis

### 5.1. LH Concentration Data Analysis

The results of LPS treatments on the concentrations of LH in all types of mediums were examined by two-way analysis of variance (ANOVA) (STATISTICA; Stat-Soft, Inc., Tulsa, OK, USA). The least significant differences post hoc test was used for the comparison of LH concentration between the 20 min periods of the *ex vivo* experiment within and between the groups. The Mann-Whitney *U* test was used to compare these values. All data are expressed as means ± SEM. Statistical significance was defined as *P* ≤ 0.01.

### 5.2. PCR Data Analysis

Relative gene expression was calculated using the comparative quantification option of Rotor Gene 6000 software 1.7. (Qiagen, Duesseldorf, Germany). The second differential maximum method [14] was used in this analysis to calculate reaction efficiencies and a set percentage of the maximum fluorescence value to calculate the beginning of the exponential phase. To compensate a variation in cDNA concentrations and the PCR efficiency between tubes, an endogenous control gene was assayed in each sample and used for normalization. Initially, three housekeeping genes: GAPDH, *β*-actin, and PPIC were tested. The BestKeeper was used to determine the most stable housekeeping gene, for normalizing genes of interest expression. The BestKeeper is based on the pairwise correlation analysis of all pairs of candidate genes [15] and calculates variations of all reference genes (SD (± Ct)). GAPDH was chosen as the best endogenous control gene. They had the lowest SD (± Ct) value and a good correlation coefficient with the remaining analyzed housekeeping genes.

The results are presented as relative gene expression of the target gene versus housekeeping gene, relative expression value, and median ± SEM. The significance of differences between the experimental groups was assessed by the Mann-Whitney *U* test. Statistical significance was defined as *P* ≤ 0.01.

## 6. Results

### 6.1. The *Ex Vivo* Effect of IL-1*β* on the LH Release

**Figure 1:**
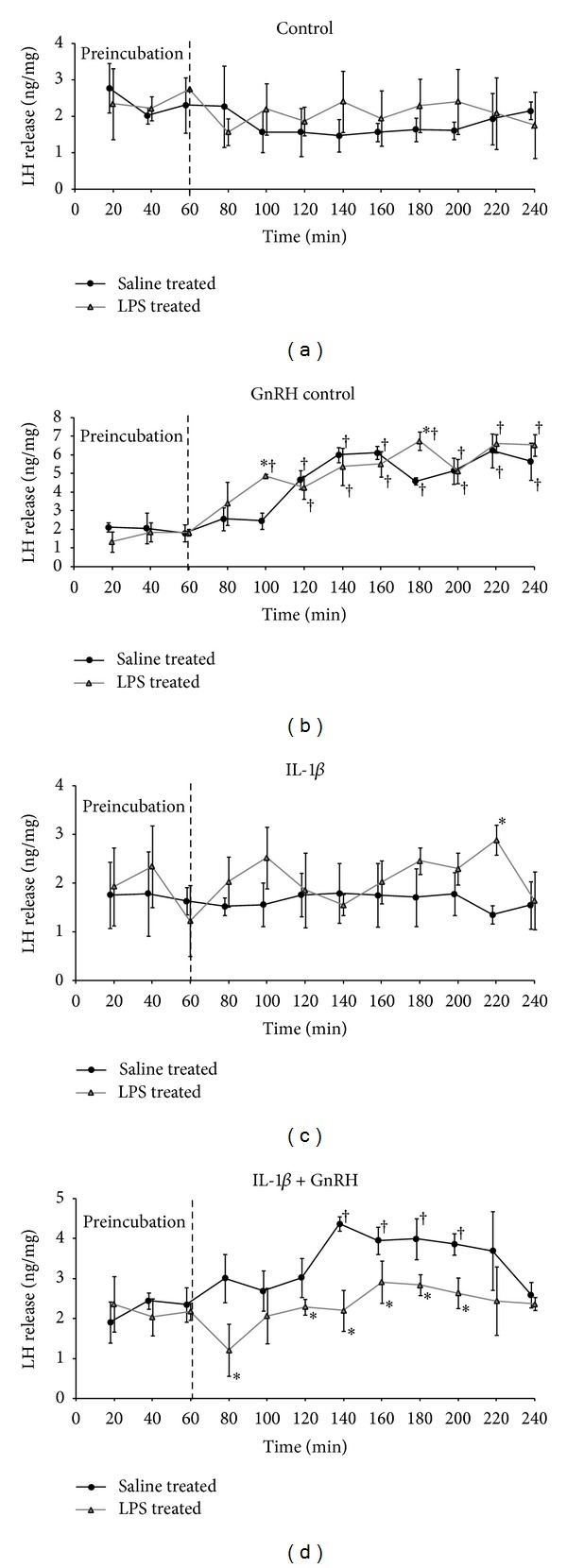
LH release from the AP explants collected from saline- and LPS- treated ewes and incubated in four types of media (control—“pure” medium 199 HEPES (a); GnRH control—medium with GnRH (100 pmol/mL) (b); IL-1*β*—medium with IL-*β* (100 pg/mL) (c); GnRH + IL-1*β* medium with GnRH (100 pmol/mL) and IL-*β* (100 pg/mL) (d)). Each curve represents median ± SEM release of LH during the consecutive 20 min periods of incubation. ^†^ *P* ≤ 0.01 (cross indicates values that differ significantly from the median LH release in the same group during preincubation period according to the Mann-Whitney *U* test) *P ≤ 0.01 (asterisk indicates values that differ significantly from the saline- treated group according to the Mann-Whitney *U* test).

In the explants collected from saline and LPS-treated ewes, the GnRH significantly (*P* ≤ 0.01) stimulated LH release, and there were no important differences in their response to GnRH (Figure 1). IL-1*β* lowered (*P* ≤ 0.01) GnRH-induced release of LH both in saline- and LPS-treated groups (Table 1). However, LPS-induced inflammation potentiated the IL-1*β*-mediated reduction of LH secretion from the AP explants. The effect of IL-1*β* was stronger (*P* ≤ 0.01) in organs collected from LPS-treated ewes where the GnRH-induced rise of LH was completely abolished than in explants from saline- injected ewes where temporary increase in the release of LH was observed (Figure 1).

### 6.2. Effect of IL-1*β* on LH-*β*, GnRH-R, and IL-1R1 Gene Expressions in the AP Explants

**Figure 2:**
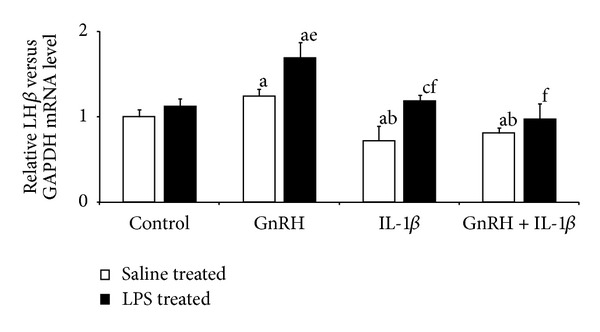
Inhibitory effect of IL-1*β* on LH*β* gene expression in the AP explants collected from saline- and LPS-treated ewes and incubated in four types of media (control—“pure” medium 199 HEPES; GnRH—medium with GnRH (100 pmol/mL); IL-1*β*—medium with IL-*β* (100 pg/mL); GnRH + IL-1*β* medium with GnRH (100 pmol/mL) and IL-*β* (100 pg/mL)). Data are presented as a median value ± SEM. a,b,c—*P* ≤ 0.01 (indicating values that differ significantly from the control, GnRH, and IL-1*β* groups of saline-treated explants, respectively, according to the Mann-Whitney *U* test) and e,f—*P* ≤ 0.01 (indicating values that differ significantly from the control and GnRH groups of LPS-treated explants, respectively, according to the Mann-Whitney *U* test).

**Figure 3:**
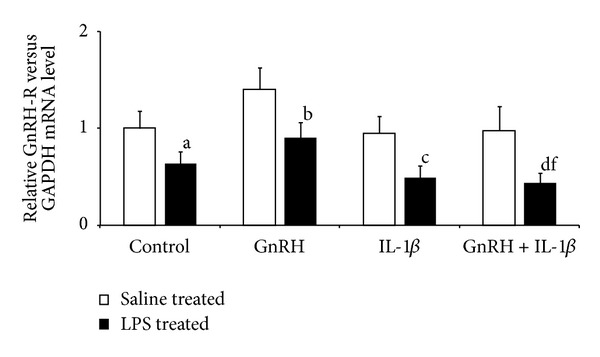
GnRH-R gene expression in the AP explants collected from saline- and LPS-treated ewes and incubated in four types of media (control—“pure” medium 199 HEPES; GnRH—medium with GnRH (100 pmol/mL); IL-1*β*—medium with IL-*β* (100 pg/mL); GnRH + IL-1*β* medium with GnRH (100 pmol/mL) and IL-*β* (100 pg/mL)). Data are presented as a median value ± SEM. a,b,c,d—*P* ≤ 0.01 (indicating values that differ significantly from the control, and GnRH, IL-1*β*, GnRH + IL-1*β* groups of saline-treated explants, respectively, according to the Mann-Whitney *U* test) and f—*P* ≤ 0.01 (indicating values that differ significantly from the GnRH group of LPS-treated explants, respectively, according to the Mann-Whitney *U* test).

In the AP explants collected from saline-treated ewes, GnRH (*P* ≤ 0.01) stimulated LH-*β* gene expression (median exp. 1.25 ± 0.08) compared to the control group (median exp. 1 ± 0.09). On the other hand, IL-1*β* decreased (*P* ≤ 0.01) the level of LH-*β* mRNA when added alone (median exp. 0.72 ± 0.17) and together with GnRH (median exp. 0.81 ± 0.06) compared to both control- and GnRH-treated groups. In AP explants collected from LPS-treated animals, GnRH also (*P* ≤ 0.01) stimulated LH-*β* gene expression (median exp. 1.69 ± 0.18) compared to the control group (median exp. 1.12 ± 0.09). However, IL-1*β* prevented the GnRH-induced increase of LH*β* gene expression (Figure 2). There was no effect of GnRH on its receptor gene expression in AP explants. However, GnRH-R mRNA level was lower (*P* ≤ 0.01) in APs collected from LPS-treated compared to saline-treated animals (Figure 3). IL-1*β* only affected the GnRH-R gene expression in explants collected from LPS-treated animals. The level of GnRH-R mRNA (median exp. 0.43 ± 0.1) was lower (*P* ≤ 0.01) in APs treated with IL-1*β* and GnRH compared to group incubated only with GnRH (median exp. 0.9 ± 0.16).

**Figure 4:**
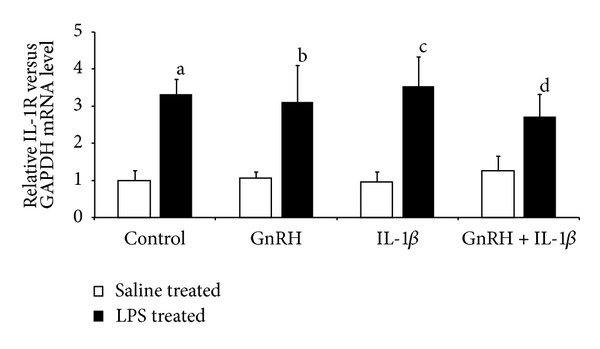
Stimulatory effect of LPS pretreatment on IL-1 type I receptor gene expression in the AP explants collected from saline- and LPS-treated ewes and incubated in four types of media (control—“pure” medium 199 HEPES; GnRH—medium with GnRH (100 pmol/mL); IL-1*β*—medium with IL-*β* (100 pg/mL); GnRH + IL-1*β* medium with GnRH (100 pmol/mL) and IL-*β* (100 pg/mL)). Data are presented as a median value ± SEM. a,b,c,d—*P* ≤ 0.01 (indicating values that differ significantly from the control, GnRH, IL-1*β*, and GnRH + IL-1*β* groups of saline-treated explants, respectively, according to the Mann-Whitney *U* test).

IL-1*β* did not affect its type I receptor gene expression in AP explants from control- and LPS-treated animals. However, the level of IL-1R1 mRNA was significantly (*P* ≤ 0.01) higher in the LPS-treated compared to saline-treated explants (Figure 4).

## 7. Discussion 

The results of our *ex vivo* studies prove that IL-1*β* is a potent downregulator of LH secretion from the pituitary and suggest that this direct action of interleukin could have a profound effect on the suppression of LH release occurring during an inflammatory state. However, obtained results are contrary to the previous *in vitro* experiments. The study performed on the pituitary cells collected from 8- to 14-month-old wethers showed that both IL-1*α* and IL-1*β* exhibited stimulatory effect on release of LH *in vitro* [16]. The results of studies carried out on cultured rat pituitary cells showed that IL-1*β* affects the secretory activity of these cells at the dose dependent manner, inhibiting FSH secretion and stimulating LH secretion [17]. Different results of our studies and cited reports could be partially due to different *in vitro* model. The present *ex vivo* studies were performed on the AP explants where cells retain the structure of the gland. That allows preserving many of intracellular interaction not available in primary culture of the pituitary. These different *in vitro* models may be crucial to explain these contradictory data, because the inhibitory action of IL-1*β* on LH secretion does not have to be an effect of its direct action on gonadotropes. These cells form multiple connections to other pituitary cells such as lactotrophs allowing cell-to-cell communication in the form of adherens junctions [18] and gap junction [19]. The presence of lactotrophs in the incubated AP explants could have a profound impact on the obtained results. It was previously reported that proinflammatory IL-1*β*, IL-6, and tumor necrosis factor *α* (TNF*α*) stimulate prolactin release directly at the rat pituitary gland [20, 21]. In turn, *in vitro* study showed that prolactin suppressed LH secretion from cultured pituitary fragments and reduced their responsiveness to GnRH [22]. This modulatory effect of prolactin on LH release may occur through the prolactin receptors existing in the gonadotropes. The study performed on sheep showed that pituitary gonadotropes exhibit expression of prolactin receptor enabling a paracrine communication between these cells and prolactin secreting lactotrophs [23]. Therefore, it is possible that IL-1*β* could suppress LH release from the AP explants indirectly via induction of prolactin secretion which in turn downregulates LH secretion. The effect of IL-1*β* on gonadotropes could be also mediated via folliculostellate cells. Folliculostellate cells play an important role as a source of paracrine factors that act locally to modulate pituitary responses to hypothalamic and peripheral signals. One of the paracrine factors that could mediate the IL-1*β* signal from folliculostellate cells to gonadotropes could be IL-6. It was found that IL-1*β* indirectly modulates the anterior pituitary cells functioning via stimulating IL-6 production from folliculostellate cells [24]. In turn, IL-6 is known as a modulator of LH release and gonadotropes response to GnRH-stimulation. However, the effect of IL-6 on pituitary gonadotropes is still ambiguous. The *in vitro* study showed that IL-6 significantly suppressed GnRH-stimulated LH release from male rats dispersed pituitaries throughout the dose range but did not influence basal LH release. In dispersed pituitaries from proestrus female rats IL-6 had no effect on basal or GnRH-stimulated LH release [25]. The other study showed stimulatory effect of IL-6 treatment on LH release from the AP cells *in vitro *[26].

Our study showed that the pituitaries kept their “memory” of the events triggered by LPS exposure, and this affected AP explants activity during *in vitro* culture. The analysis of GnRH-R gene expression showed that the transcription of this receptor was lower in the APs from LPS-treated animals compared with explants collected from control one. This fully supports the results of studies carried out on anestrous ewes when an immune stress decreased the amount of GnRH-R mRNA in the AP [1]. The *in vivo* study performed on ovariectomized ewes showing that bacterial endotoxin decreased the pituitary responsiveness to GnRH stimulation [27]. It was found that LPS suppressed the amplitude of LH pulses induced by artificial GnRH pulses. It is hard to compare the results of *in vivo* and *in vitro* studies. Our results suggest that the used concentration of GnRH was sufficient to stimulate LH secretion even in the pituitary cells with decreased expression of GnRH-R. The immune stress decreased GnRH-R mRNA content but did not decrease the pituitary responsiveness to GnRH stimulation. GnRH stimulated LH release from explants collected from both LPS- and saline-treated animals. The lack of direct connection between the expression of GnRH-R and LH release from pituitary did not surprise. The study performed on ovariectomized ewes showed that the magnitude and direction of the change in GnRH-R number do not account for the changes in pituitary responsiveness to GnRH [28]. It is noteworthy that although IL-1*β* did not affect the GnRH-R gene expression in pituitaries collected from saline-treated ewes, IL-1*β* affected the GnRH-R mRNA level in explants collected from LPS-treated animals. That suggests that inflammatory challenges increased pituitary sensitivity to this cytokine action.

It was found that the potency of IL-1*β* to affect the LH secretion from pituitary cells seems to be dependent upon the physiological status of the animal before collection of organs. The effect of IL-1*β* treatment on LH release was stronger in pituitaries collected from LPS-treated ewes LH release in APs from LPS-treated ewes from the beginning of the experiment. In APs explants collected from control animals, IL-1*β* only reduced the GnRH-induced release of LH during the first 200 min of incubation. However, at the end of the experiment, the effect of IL-1*β* was considerable. The increased sensitivity of pituitary cells collected from LPS-treated ewes on IL-1*β* action seems to result directly from higher expression of IL-1R1 in these cells. It was found that the level of mRNA encoding IL-1R1 in LPS-treated animals was significantly higher compared to saline-treated ewes. The presence of membrane IL-1R1 is essential for a tissue response on IL-1*β* action. Although IL-1R1-deficient mice show no abnormal phenotype in health and exhibit normal homeostasis, they do exhibit reduced responses to challenge with inflammatory agents [29]. IL-1RI-deficient mice also show an attenuated inflammatory response compared with wild-type mice [30]. The stimulatory effect of immune stress on IL-1R1 expression in the brain tissue has been previously described both in rats and sheep [31, 32]. However, the effect of endotoxin on the expression of IL-1R1 in the pituitary is not clear. The *in vitro* study on the mouse AtT-20 pituitary tumor cells showed that direct LPS treatment increases the number of IL-1R1 in a dose-dependent manner [33]. The studies carried out on mice [34] and sheep [31] also reported the stimulating effect of LPS on IL-1R1 mRNA. However, the other studies suggested the inhibitory effect of LPS on IL-1R1 gene expression in the pituitary [35, 36], In the present study observed elevation of sheep IL-1R1 mRNA does not have to be an effect of direct action of LPS on pituitary cells. It could be caused by stress of inflammatory response. Stress is a profound upregulator of IL-1R1 expression. The study performed on mice showed that ether-laparotomy stress resulted in a selective increase in pituitary IL-1 receptors. Moreover, an intraperitoneal injection of rat/human CRF mimicked the effects of stress and resulted in a dramatic increase of IL-1 receptor level in the pituitary [35].

In conclusion, the study showed that IL-1*β* is a potent modulator of LH secretion at the pituitary level. However, the potency of IL-1*β* to affect the secretory activity of gonadotropes seems to be dependent upon the physiological status of animals. We also discovered that the pituitaries “memory” of the events triggered by LPS exposure seems to result from different expression of cytokines receptors. This suggests that inflammatory stress affects the activity of the pituitary in a prolonged manner, and it could affect its function even for many hours after deprivation of inflammatory signals.

## Figures and Tables

**Table 1:** Summary release of LH from the AP explants collected from saline and LPS-treated ewes during the 3 h incubation period.

Group	Concentration of LH (ng/mg)	
	Saline treated	LPS treated
Control	15.8 ± 3.6	18.5 ± 5.3
GnRH control	43.5 ± 4.1^a^	48.5 ± 4.6^e^
IL-1*β*	14.8 ± 3.2	19.3 ± 4.1
IL-1*β* + GnRH	31.2 ± 3.0^abc^	21.0 ± 3.4^df^

^a,b,c,d^ *P* ≤ 0.01 (indicating values that differ significantly from the control, GnRH control, IL-1*β*, and IL-1*β* + GnRH groups of saline-treated explants, respectively, according to the Mann-Whitney *U*-test).

^e,f^ *P* ≤ 0.01 (indicating values that differ significantly from the control and GnRH control groups of LPS-treated explants, respectively, according to the Mann-Whitney *U*-test).

Data are presented as a median value ± SEM.
